# The Relationship Between Lichen Planus and Thyroid Disorders in an Iranian Population: A Case‐Control Study

**DOI:** 10.1002/hsr2.73273

**Published:** 2026-09-19

**Authors:** Safoura Shakoei, Helya Bolouki Azari, Arman Soleimany, Adnan Mastalizadeh, Maryam Daneshpazhooh

**Affiliations:** ^1^ Department of Dermatology, Imam Khomeini Hospital Complex Tehran University of Medical Sciences (TUMS) Tehran Iran; ^2^ Department of Dermatology, Razi Hospital Tehran University of Medical Sciences (TUMS) Tehran Iran; ^3^ Sleep Breathing Disorders Research Center (SBDRC) Tehran University of Medical Sciences Tehran Iran; ^4^ School of Medicine Tehran University of Medical Sciences (TUMS) Tehran Iran; ^5^ Autoimmune Bullous Diseases Research Center, Department of Dermatology Tehran University of Medical Sciences (TUMS) Tehran Iran

**Keywords:** cutaneous lichen planus, lichen planopilaris, lichen planus, thyroid diseases

## Abstract

**Background and Aims:**

Previous research investigated the connection between lichen planus (LP) and thyroid disorders due to their autoimmune characteristics; however, the correlation remains unclear. This study investigates the relationship between thyroid disorders and specific subtypes of LP, particularly cutaneous lichen planus (CLP) and lichen planopilaris (LPP), to determine the need for thyroid screening in LP patients.

**Methods:**

This case‐control study at Razi Hospital's skin clinic (2023–2024) involved 70 LP patients and 70 controls. A dermatologist confirmed the diagnosis via clinical evaluation and biopsy. Medical histories, medication, and BMI were collected. Serological tests for thyroid disorders were conducted, and patients were categorized accordingly. Data analysis was performed using SPSS version 22.

**Results:**

The study shows a significant link between LP and thyroid disorders (*p* = 0.007), consistent across LP subtypes (CLP and LPP). LP patients had higher TSH (*p* = 0.008) and anti‐TPO antibody levels (*p* < 0.001), while free T4 levels were lower but not statistically significant (*p* = 0.38). Anti‐TPO antibody levels were significantly elevated in both LP subtypes (*p* = 0.01 in CLP and *p* = 0.002 in LPP) compared to controls. Age, gender, BMI, and T3 levels did not significantly correlate with LP. Longer LP duration was associated with higher TSH levels (*p* = 0.05).

**Conclusion:**

There is a significant relationship between LP and thyroid disorders, particularly hypothyroidism. These findings advocate for regular thyroid function monitoring in LP patients to facilitate early detection and management of thyroid issues.

## Key Point

Our results show a strong connection between lichen planus and thyroid disorders, especially hypothyroidism, regardless of the LP type. This highlights the importance of regular thyroid function checks in LP patients for early detection and management.

## 1 Introduction

**Figure 1:**
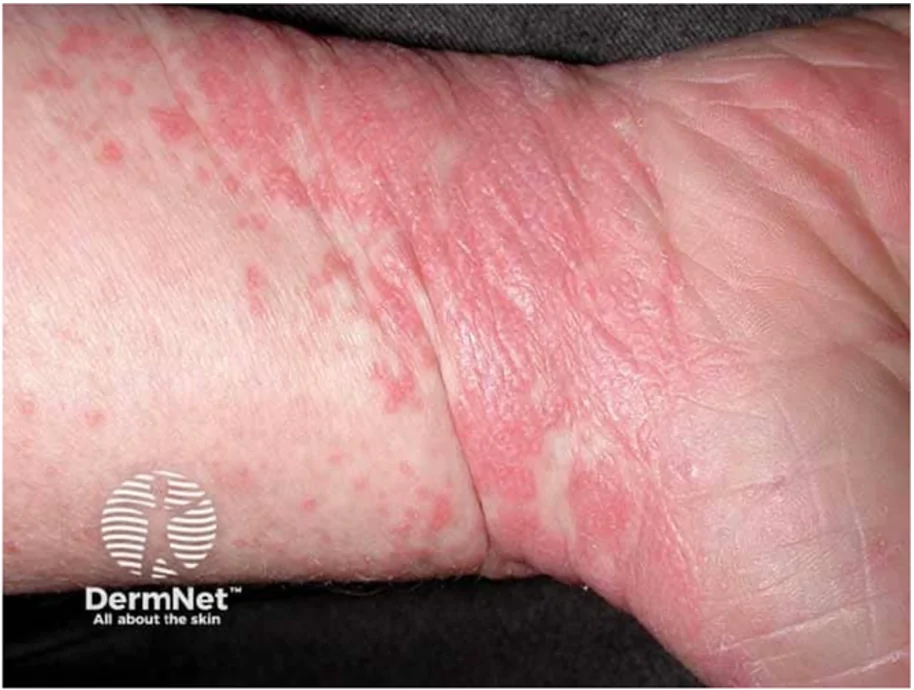
Papular lichen planus coalescing into plaques on the wrist. Image sourced from DermNet NZ (Papular lichen planus image), used under the Creative Commons Attribution–Noncommercial–NoDerivs license.

**Figure 2:**
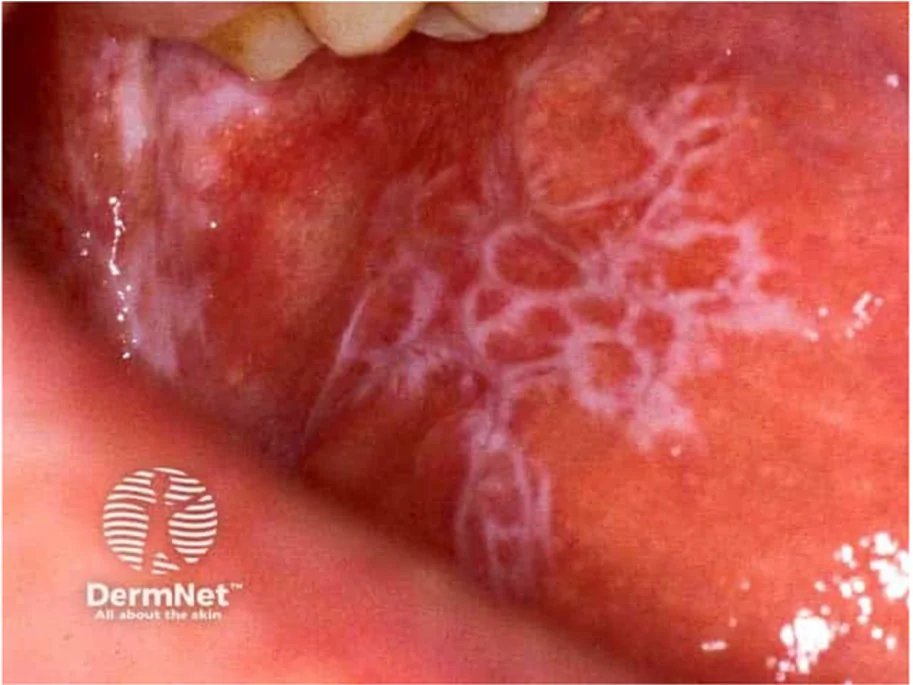
Reticulate buccal mucosal lichen planus (OLP). Image sourced from DermNet NZ (https://dermnetnz.org/imagedetail/3051-oral-lichen-planus), used under the Creative Commons Attribution–Noncommercial–NoDerivs license.

**Figure 3:**
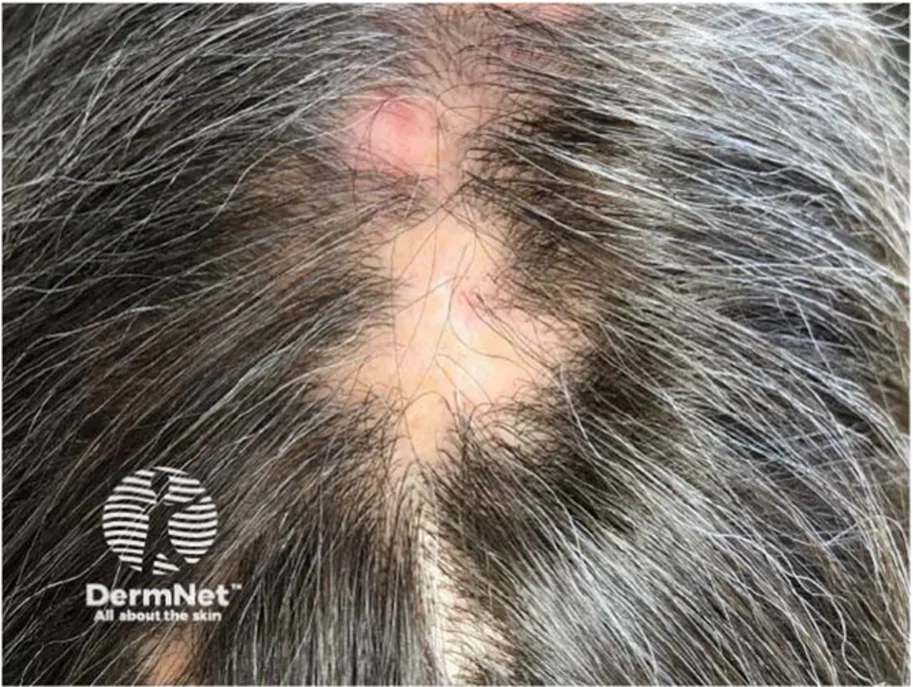
Lichen planopilaris. Image sourced from DermNet NZ (https://dermnetnz.org/imagedetail/16040-lichen-planopilaris), used under the Creative Commons Attribution–Noncommercial–NoDerivs license.

Lichen planus (LP) is an inflammatory disorder commonly observed in middle‐aged individuals. LP has three major subtypes: Cutaneous Lichen Planus (CLP) (Figure 1), Mucosal or Oral Lichen Planus (OLP) (Figure 2), and Follicular Lichen Planus, or Lichen Planopilaris (LPP) (Figure 3) [1]. The prevalence of CLP in adults is 0.2%–1%, which is more common than OLP in most studied populations [2, 3]. OLP is more frequent in women, but there is no gender difference in the prevalence of CLP [4, 5, 6]. Almost two‐thirds of patients with LP are between 30 and 60 years old [7, 8].

Autoimmune thyroid diseases (AITD) include Graves’, Hashimoto's thyroiditis, and non‐endemic goiters. The first two are among the most common causes of thyroid dysfunction. Studies have shown that patients with AITD are more vulnerable to skin diseases such as alopecia areata, generalized itching, chronic urticaria, vitiligo, and myxedema [9]. On the other hand, several autoimmune diseases, such as myasthenia gravis, Sjögren's syndrome, ulcerative colitis, psoriasis, thymoma, lupus erythematosus, and celiac disease, are related to LP [10, 11]. Hence, AITD and LP may be correlated.

Several researchers have assessed the association between LP and thyroid dysfunctions. The results of certain studies suggest a significant relationship between elevated anti‐thyroid peroxidase (anti‐TPO) antibody levels in the blood and a greater likelihood of experiencing erosive OLP, presenting the anti‐TPO antibody levels as a valuable indicator of underlying thyroid abnormalities in OLP patients. However, alternative studies have found no significant correlation between hypothyroidism and the incidence of OLP [12, 13]. The incidence of Hashimoto's thyroiditis in individuals with OLP, particularly among female patients, is reported to be notably higher compared to the general populace, and female patients demonstrate elevated titers of Hashimoto's thyroiditis autoantibodies in comparison to their male counterparts [14]. The most recent systematic review and meta‐analysis on the prevalence of autoimmune disorders among OLP patients and their association indicate the potential for OLP‐affected individuals to acquire an autoimmune condition that makes them prone to auto‐aggression toward various targets [15]. In addition, patients with thyroid disorders are shown to be more susceptible to developing OLP and oral lichenoid lesions [16]. The findings of a previous study indicated that LPP was correlated with an increased risk of several conditions, such as systemic lupus erythematosus, psoriasis, rheumatoid arthritis, LP, atopic dermatitis, allergic rhinitis, nonmelanoma skin cancer, vitamin D deficiency, and thyroid diseases (hyperthyroidism, hypothyroidism, and thyroiditis) [17]. However, some studies have yielded controversial results, such as indicating a lower prevalence of thyroid disorders among patients with OLP [18].

Based on previous scholarly debates, there is a current gap in understanding LP and thyroid disease associations, and so far, the topic is still a matter of debate.

The objective of this study was to evaluate the association between thyroid disorders and LP, with a particular focus on its subtypes, CLP and LPP, in an Iranian population. Additionally, we aimed to assess whether patients with LP may benefit from routine thyroid function screening to reduce or prevent complications.

## 2 Methods

### 2.1 Study Design

This case‐control study was conducted at the dermatology clinic of Razi Hospital, Tehran, Iran, between 2023 and 2024. The study included 70 patients diagnosed with LP, including CLP and LPP, and 70 age‐ and sex‐matched controls.

Patients with LP were consecutively recruited from the dermatology clinic during the study period. The diagnosis of LP was confirmed by an experienced dermatologist based on clinical findings, and histopathological examination was performed when necessary.

The sample size was calculated based on previously reported prevalence rates of thyroid disorders in patients with LP (72.4%) and in healthy controls (49.4%). With a Significance (α) level of 0.05 and a Power of 80%, the minimum number of participants required was 63 per group. To account for dropouts, 70 participants were included per condition.

### 2.2 Participants

Patients diagnosed with LP at 18 years old or older were included in this study. Exclusion criteria were [1]: history of autoimmune or systemic inflammatory diseases (e.g., psoriasis, vitiligo, alopecia areata, Bechet's disease, kidney disease) [2]; use of medications affecting thyroid function (including Amiodarone, Propranolol, Tamoxifen, Phenytoin, Carbamazepine, Dopamine, Salicylate, Non‐steroidal anti‐inflammatory drugs, and Corticosteroids) [3]; pregnancy [4]; history of hormone replacement therapy or concurrent use of the combined oral contraceptive pills.

The control group consisted of individuals attending the same clinic for cosmetic procedures (e.g., botulinum toxin injection, fillers, or benign lesion removal), with no history of LP or known thyroid disease.

### 2.3 Data Collection

Patient medical histories, medications, and body mass index (BMI) were collected. Serological tests were applied to investigate thyroid disorders using the serum levels of thyroid‐stimulating hormone (TSH), free thyroxine (T4), total triiodothyronine (T3), and anti‐TPO antibody. We used the Electrochemiluminescence (ECL) method and the ROCHE kit to measure TSH and total T3. To measure anti‐TPO antibodies and free T4, an enzyme‐linked immunosorbent assay (ELISA) was used. Patients were diagnosed with hypothyroidism, hyperthyroidism, subclinical hypothyroidism, subclinical hyperthyroidism, or positive anti‐TPO antibody cases according to the diagnostic criteria and the standard test values (TSH between 0.270 and 4.20 μUI/mL, free T4 between 0.8 and 2.0 ng/dL, total T3 between 1.3 and 3.1 nmol/L, and positive anti‐TPO antibody > 39.2 AU/ML).

### 2.4 Statistical Analysis

Statistical analysis was performed using SPSS software (version 22.0; IBM Corp., Armonk, NY, USA). All analyses were pre‐specified, and no exploratory subgroup analyses were performed.

Continuous variables were assessed for normality using the Kolmogorov–Smirnov test and are presented as mean ± standard deviation (SD). Although some continuous variables showed deviations from normality, independent‐samples *t*‐tests were retained for between‐group comparisons given the moderate sample size and the known robustness of the *t*‐test to departures from normality. Mean differences (MDs) with 95% confidence intervals (CIs) were reported. Categorical variables were presented as frequencies. They were compared using the chi‐square test or Fisher's exact test, as appropriate, and odds ratios (ORs) with 95% CIs were calculated for categorical outcomes. A significance level of α = 0.05 was used for all analyses.

### 2.5 Ethical Considerations

All procedures were conducted in accordance with the ethical standards of the institutional and national research committee and with the Declaration of Helsinki.

The study protocol was approved by the Ethics Committee of Tehran University of Medical Sciences (IR. TUMS. IKHC. REC.1399.245). Written informed consent was obtained from all participants prior to enrollment. All data were anonymized to ensure participant confidentiality.

## 3 Results

In this study, 70 patients with LP and 70 controls participated. The case and control groups were matched by age and gender. The average age of all participants was 46.1 ± 12.6 years, with 46 men (32.9%) and 94 women (67.1%). Descriptive statistics for other variables are shown in Table 1. Anti‐TPO antibody levels were higher in patients with LP (*p* < 0.001). TSH levels were higher in patients with LP, with a mean difference of 1.59 µIU/mL (95% CI: 0.20–2.98), suggesting a potentially clinically relevant elevation (*p* = 0.008).

**Table 1:** Clinical and laboratory results of lichen planus patients and healthy controls.

Variables	Lichen Planus patients (*n* = 70)	Control group (*n* = 70)	Mean difference (95% CI)	*p* value
Age, years (Mean ± SD)	46.23 ± 12.5	46.1 ± 12.7	0.13 (−4.05 to 4.31)	0.92^a^
Gender, no (%)			—	> 0.99^b^
Male	23 (32.9%)	23 (32.9%)		
Female	47 (67.1%)	47 (67.1%)		
BMI (Mean ± SD)	27.9 ± 4.4	26.9 ± 4.0	1.00 (−0.39–2.39)	0.15^a^
TSH (µIU/mL) (Mean ± SD)	4.46 ± 3.96	2.91 ± 3.51	1.55 (0.31–2.79)	**0.008** a
Free T4 (ng/dL) (Mean ± SD)	1.10 ± 0.75	1.19 ± 0.43	−0.09 (−0.292–0.112)	0.38^a^
Total T3 (nmol/L) (Mean ± SD)	1.96 ± 0.39	2.05 ± 0.47	−0.09 (−0.233–0.053)	0.25^a^
Anti‐TPO Ab (AU/mL) (Mean ± SD)	160.8 ± 68.3	106.5 ± 37.2	54.3 (36.1–72.5)	**< 0.001** a

*Note:* Bold *p* values indicate statistical significance.

Abbreviations: Anti‐TPO Ab, Anti‐Thyroid Peroxidase Antibody; BMI, Body mass index; CI, Confidence Interval; T3, Triiodothyronine; T4, Thyroxine; TSH, Thyroid‐stimulating hormone.

^a^ Independent‐samples *t*‐test.

^b^ Chi‐square test.

Additionally, Free T4 levels were lower in the patient group; however, this difference was not statistically significant (*p* = 0.38). Other variables showed no significant differences between the patients and controls (Table 1).

Regarding the association between the duration of LP and the study variables (age, gender, height, weight, BMI, TSH, free T4, total T3, and anti‐TPO Ab), only TSH exhibits a significant relationship with the duration of LP, showing a positive correlation coefficient of 0.24 (*p* = 0.05) (Table 2).

**Table 2:** Correlation between duration of lichen planus and clinical/laboratory variables.

Variable	Correlation coefficient (r)	*p* value
Age	0.05	0.68^a^
Height	−0.02	0.84^a^
Weight	−0.13	0.29^a^
BMI	−0.02	0.84^a^
TSH (µIU/mL)	0.24	**0.05** a
Free T4 (ng/dL)	−0.11	0.35^a^
Total T3 (nmol/L)	−0.05	0.68^a^
Anti‐TPO (AU/mL)	−0.01	0.95^a^

*Note:* Bold *p* values indicate statistical significance.

Abbreviations: Anti‐TPO Ab, Anti‐Thyroid Peroxidase Antibody; BMI, Body mass index; T3, Triiodothyronine; T4, Thyroxine; TSH, Thyroid‐stimulating hormone.

^a^ Pearson's correlation coefficient.

A comparison of study variables in CLP and LPP patients with the control group is detailed in Table 3. The female‐to‐male ratio was higher in LPP than in CLP. TSH levels were significantly higher in patients with CLP than in controls (mean difference [MD] = 1.59 µIU/mL, 95% CI: 0.20–2.98, *p* = 0.002), but there was no significant difference in the LPP group. Anti‐TPO antibody levels were significantly higher in both CLP and LPP patients compared to controls (CLP: MD = 49.5 AU/mL, 95% CI: 27.0–72.0, *p* = 0.01; LPP: MD = 64.4 AU/mL, 95% CI: 37.9–90.9, *p* = 0.002). Free T4 levels were lower in patients with CLP and LPP than in controls; however, these differences were not statistically significant (*p* = 0.60 and *p* = 0.48, respectively). There were no significant differences in other variables between the patients and control groups (Table 3).

**Table 3:** Clinical and laboratory results of cutaneous lichen planus and lichen planopilaris patients compared to the control group.

Variables	CLP (*n* = 43)	LPP (*n* = 27)	Control group (*n* = 70)	MD (CLP−Control) [95% CI]	*p* value (CLP vs. Control)	MD (LPP−Control) [95% CI]	*p* value (LPP vs. Control)
Age, years (Mean ± SD)	47 ± 13	45.1 ± 11.9	46.1 ± 12.7	0.9 [−4.0, 5.8]	0.71	−1.0 [−6.4, 4.4]	0.73^a^
Gender, No (%)				—	0.33	—	0.16^b^
Male	18 (41.9%)	5 (18.5%)	23 (32.9%)				
Female	25 (41.9%)	22 (81.5%)	47 (67.1%)				
BMI (Mean ± SD)	28.3 ± 5	27.4 ± 3.3	26.9 ± 4.0	1.4 [−0.36, 3.16]	0.11	0.5 [−1.07, 2.07]	0.59^a^
TSH (µIU/mL) (Mean ± SD)	4.5 ± 3.77	4.4 ± 4.32	2.91 ± 3.51	1.59 [0.20, 2.98]	**0.002**	1.49 [−0.33, 3.31]	0.34^a^
Free T4 (ng/dL) (Mean ± SD)	1.11 ± 0.95	1.07 ± 0.24	1.19 ± 0.43	−0.08 [−0.38, 0.22]	0.60	−0.12 [−0.26, 0.02]	0.48^a^
Total T3(nmol/L) (Mean ± SD)	1.92 ± 0.35	2.04 ± 0.43	2.05 ± 0.47	−0.13 [−0.28, 0.02]	0.12	−0.01 [−0.21, 0.19]	0.90^a^
Anti‐TPO (AU/mL) (Mean ± SD)	156 ± 69.5	170.9 ± 66.4	106.5 ± 37.2	49.5 [27.0, 72.0]	**0.01**	64.4 [37.9, 90.9]	**0.002** a

*Note:* Bold *p* values indicate statistical significance.

Abbreviations: Anti‐TPO Ab, Anti‐Thyroid Peroxidase Antibody; BMI: Body mass index; CI, Confidence Interval; CLP, Cutaneous Lichen Planus; LPP, Lichen Planopilaris; MD, Mean Difference; T3: Triiodothyronine; T4, Thyroxine; TSH, Thyroid‐stimulating hormone.

^a^ Independent‐samples *t*‐test.

^b^ Chi‐square test.

We investigated the correlation between thyroid disorders and LP patients and the control group and found that 36 of 140 participants (25.7%) had a thyroid disorder—25 in the LP group (35.7%) and 11 in the control group (15.7%). Patients with LP had a higher odds of thyroid disorders than controls (OR = 2.98, 95% CI: 1.33–6.66, *p* = 0.007). The odds of hypothyroidism were markedly higher in LP patients (OR = 12.86, 95% CI: 1.60–102.4, *p* = 0.003). Out of the 12 people with hypothyroidism, 11 also had LP disease (7 CLP and 4 LPP). Also, the number of people with hyperthyroidism in the control group was higher than in the LP group, but this was not statistically significant (*p* = 0.62). In addition, while the number of people with subclinical hypothyroidism in LP patients was almost twice that of the control group, this was not a significant finding (*p* = 0.19). The study revealed no statistically significant correlation between subclinical hyperthyroidism or positive anti‐TPO antibody and LP disease (Table 4).

**Table 4:** Prevalence and association of thyroid disorders with lichen planus compared to controls.

Variables	Lichen Planus (*n* = 70)	Control group (*n* = 70)	*p* value	OR (95% CI)
Any thyroid disorder, no (%)	25 (35.7%)	11 (15.7%)	**0.007** a	2.98 (1.33–6.66)
Hypothyroidism, no (%)	11 (15.7%)	1 (1.4%)	**0.003** a	12.86 (1.60–102.4)
Hyperthyroidism, no (%)	1 (1.4%)	3 (4.3%)	0.62^b^	0.32 (0.03–3.19)
Subclinical hypothyroidism, no (%)	11 (15.7%)	6 (8.6%)	0.20^a^	1.99 (0.69–5.73)
Subclinical hyperthyroidism, no (%)	2 (2.9%)	1 (1.4%)	> 0.99^b^	2.03 (0.18–22.9)

*Note:* Bold *p* values indicate statistical significance (*p* < 0.05).

Abbreviations: CI, confidence interval; OR, odds ratio.

^a^ Chi‐square test.

^b^ Fisher's exact test.

Regarding the type of LP, thyroid disorders were observed in 34.9% of CLP patients and 37.0% of LPP patients, with no statistically significant difference between the two groups (*p* = 0.85). No significant associations were found between the type of LP and specific thyroid conditions (Table 5). Moreover, Anti‐TPO positivity was observed in 20.9% of CLP patients and 14.8% of LPP patients, with no significant difference between groups (OR = 1.52, 95% CI: 0.42–5.44; *p* = 0.52) (Table 5).

**Table 5:** Prevalence of thyroid disorders according to type of lichen planus.

Variables	CLP (*n* = 43) *n* (%)	LPP (*n* = 27) *n* (%)	OR (95% CI)	*p* value
Any thyroid disorder	15 (34.9)	10 (37.0)	0.91 (0.34–2.41)	0.855^a^
Hypothyroidism	7 (16.3)	4 (14.8)	1.12 (0.29–4.27)	> 0.99^b^
Hyperthyroidism	1 (2.3)	0 (0)	—	> 0.99^b^
Subclinical hypothyroidism	6 (14.0)	5 (18.5)	0.72 (0.19–2.66)	0.739^b^
Subclinical hyperthyroidism	1 (2.3)	1 (3.7)	0.62 (0.04–10.6)	> 0.99^b^
Anti‐TPO positive	9 (20.9)	4 (14.8)	1.52 (0.42–5.44)	0.522^a^

Abbreviations: CI, confidence interval; OR, odds ratio.

^a^ Chi‐square test.

^b^ Fisher's exact test.

Also, the female‐to‐male ratio is higher in LP patients with thyroid disorders than in those without thyroid disorders, but this is not statistically significant (*p* = 0.08). Moreover, LP patients with thyroid disorders tend to have longer disease durations, but the reported values do not indicate a significant correlation.

## 4 Discussion

Our study presents a significant association between LP and thyroid disorders, specifically hypothyroidism. LP patients had significantly higher TSH and anti‐TPO antibody levels than healthy controls. Free T4 levels were lower among LP patients; however, this finding did not reach statistical significance. Also, there was a significant positive correlation between disease duration and higher TSH levels, suggesting that LP patients may exhibit a cumulative effect on thyroid function over time, or vice versa. Therefore, routine thyroid evaluations should be considered in the clinical assessment of patients with LP.

The observed association between LP and hypothyroidism can be explained by the notion of both conditions being classified as autoimmune disorders. LP is a chronic inflammatory disease mediated by T cells [19, 20, 21], and AITD, including Hashimoto's thyroiditis, involves similar immune dysregulation [22]. Chronic immune activation in LP may contribute to the production of autoantibodies, including anti‐TPO, which can damage thyroid tissue and impair hormone synthesis. Alternatively, systemic dysregulation of the immune system in individuals with hypothyroidism may predispose these individuals to the development of inflammatory skin disorders, such as LP, thereby creating a bidirectional relationship between the two conditions.

Our findings are consistent with several studies reporting an association between LP—particularly OLP—and thyroid disorders, especially autoimmune conditions such as Hashimoto's thyroiditis [9, 13, 14, 16, 18, 23, 24, 25, 26]. Previous research findings support the theory of a shared autoimmune background between patients with OLP and thyroid autoimmunity. They have concluded that OLP patients are more prone to hypothyroidism and thyroid autoimmunity [18, 25]. Similarly, studies in LPP populations have reported a higher incidence of thyroid diseases, further suggesting that thyroid dysfunction may play a role across different LP subtypes [17, 27]. Unlike our findings, Lim's retrospective study also suggested a positive association between hyperthyroidism and LP [17]. In addition, some evidence indicates that thyroid disease itself may increase the risk of developing OLP or lichenoid lesions, implying a potentially bidirectional relationship [16, 23].

The relationship between thyroid autoimmunity and the clinical expression of OLP remains complex and somewhat inconsistent across studies. While an increased prevalence of Hashimoto's thyroiditis among OLP patients—particularly in women—has been reported, this does not always appear to translate into differences in clinical severity or presentation [14]. Similarly, although some studies have observed a higher frequency of hypothyroidism in OLP patients, the clinical course of the disease may not necessarily be more severe in these individuals [26]. In contrast, M. Alikhani et al. suggest that elevated anti‐TPO antibody levels may be associated with increased disease severity, raising the possibility that thyroid autoimmunity could influence disease progression in at least a subset of patients [13].

From a broader perspective, De Porras‐Carrique et al. [15] reported in a systematic review and meta‐analysis that the coexistence of OLP and AITD has been interpreted as part of a shared autoimmune predisposition rather than a direct causal relationship. However, this interpretation is not universally supported. Some evidence shows no clear association between anti‐thyroid antibodies and OLP, despite the presence of biochemical patterns suggestive of hypothyroidism in certain patients. J. Robledo‐Sierra's study found that only 10% of OLP patients had increased anti‐thyroid antibodies, compared to 16.1% in the general population, showing no correlation between anti‐thyroid antibodies and OLP. However, many OLP patients without prior thyroid disease had markers indicative of hypothyroidism (high TSH and low FT4 levels). Interestingly, the reported expression of thyroid‐stimulating hormone receptors in OLP lesions suggests a possible localized autoimmune interaction, which may partially explain these conflicting findings [9].

The association between LP and thyroid disorders remains inconsistent across studies. Several studies have not confirmed a significant association, suggesting that this link may not be universal or may vary across LP subtypes. For instance, some studies have found no meaningful association between LPP or OLP and thyroid dysfunction, suggesting that distinct pathogenic mechanisms may be involved in these subtypes [28, 29].

Similarly, the lack of correlation between hypothyroidism and OLP in certain populations [12], together with findings that question the role of TSH and its receptor in disease pathogenesis [30], further challenges the existence of a direct or uniform connection. On the other hand, there are also reports indicating a higher prevalence of thyroid disorders and elevated anti‐thyroglobulin antibody levels in LPP patients, although without consistent alterations in standard thyroid hormone levels or anti‐TPO antibodies, suggesting a more complex or indirect relationship [31].

In our study, age and gender were not significantly linked to LP, its subtypes, or duration. Although the female‐to‐male ratio was higher among LP patients with thyroid disorders, this was not statistically significant. This contrasts with some previous reports that suggest a female predominance in both LPP and AITD, implying that gender‐related immunological differences may still play a role, even if not consistently demonstrated across all studies [14, 18, 32].

Our study faced several limitations. First, the sample size may restrict generalizability. However, as the results align with previous studies, the observed patterns are unlikely to be due to chance. These preliminary findings provide a foundation for larger‐scale studies that can more thoroughly examine these relationships. Second, due to practical constraints, we used a case‐control design rather than a randomized controlled trial or a cohort study, increasing the risk of selection and recall bias. Third, the absence of randomization introduces potential confounding factors. Lastly, numerous confounding factors, such as age, sex, medications, and co‐existing conditions, may influence thyroid function test results. The effect of these and other confounding factors on the interpretation of the relationship between independent variables and thyroid disease is highly complex. While this study controlled for age and sex to minimize confounding, future studies should consider confounding factors more comprehensively.

Despite its limitations, our study adds to the existing evidence regarding the association between LP and thyroid disease, and provides direction for future research and clinical practice. Additional studies with larger sample sizes and more rigorous designs are required to validate these findings and address current limitations.

## 5 Conclusions

In conclusion, our findings support a significant association between LP and thyroid disorders, particularly hypothyroidism. This is likely driven by shared autoimmune mechanisms. This underscores the clinical relevance of routine thyroid function monitoring in LP patients to enable early detection and management of thyroid disease and potentially improve overall patient outcomes.

## Author Contributions

**Safoura Shakoei:** conceptualization, methodology, supervision, resources, writing – review and editing, validation, funding acquisition. **Helya Bolouki Azari:** writing – review and editing, writing – original draft, project administration, investigation, methodology. **Arman Soleimany:** writing – original draft, writing – review and editing, investigation, validation, visualization. **Adnan Mastalizadeh:** conceptualization, data curation, investigation. **Maryam Daneshpazhooh:** methodology, software, data curation, formal analysis.

## Conflicts of Interest

The authors declare no conflicts of interest.

## Transparency Statement

The lead author, Safoura Shakoei, affirms that this manuscript is an honest, accurate, and transparent account of the study being reported; that no important aspects of the study have been omitted; and that any discrepancies from the study as planned have been explained.

## Data Availability

The data that support the findings of this study are available from the corresponding author upon reasonable request. “All authors have read and approved the final version of the manuscript. Safoura Shakoei had full access to all of the data in this study and takes complete responsibility for the integrity of the data and the accuracy of the data analysis.”

## References

[1] F. Gorouhi , P. Davari , and N. Fazel , “Cutaneous and Mucosal Lichen Planus: A Comprehensive Review of Clinical Subtypes, Risk Factors, Diagnosis, and Prognosis,” Scientific World Journal 2014 (2014): 1–22. 10.1155/2014/742826 PMC3929580 24672362

[2] A. S. Boyd and K. H. Neldner , “Lichen Planus,” Journal of the American Academy of Dermatology 25, no. 4 (1991): 593–619. 10.1016/0190-9622(91)70241-s 1791218

[3] L. Le Cleach and O. Chosidow , “Lichen Planus,” New England Journal of Medicine 366, no. 8 (2012): 723–732. 10.1056/NEJMcp1103641 22356325

[4] T. Axéll and L. Rundquist , “Oral Lichen Planus—A Demographic Study,” Community Dentistry and Oral Epidemiology 15, no. 1 (1987): 52–56. 10.1111/j.1600-0528.1987.tb00480.x 3467894

[5] C. Irvine , F. Irvine , and R. H. Champion , “Long‐Term Follow‐Up of Lichen Planus,” Acta Dermato‐Venereologica 71, no. 3 (1991): 242–244. 1678229

[6] C. Li , X. Tang , X. Zheng , et al., “Global Prevalence and Incidence Estimates of Oral Lichen Planus: A Systematic Review and Meta‐Analysis,” JAMA dermatology 156, no. 2 (2020): 172–181. 10.1001/jamadermatol.2019.3797 PMC6990670 31895418

[7] Z. Schwager , M. Stern , J. Cohen , and A. Femia , “Clinical Epidemiology and Treatment of Lichen Planus: A Retrospective Review of 2 Tertiary Care Centers,” Journal of the American Academy of Dermatology 81, no. 6 (2019): 1397–1399. 10.1016/j.jaad.2019.04.027 31004729

[8] G. Wagner , C. Rose , and M. M. Sachse , “Clinical Variants of Lichen Planus,” Journal der Deutschen Dermatologischen Gesellschaft 11, no. 4 (2013): 309–319. 10.1111/ddg.12031 23320493

[9] J. Robledo‐Sierra , K. Landin‐Wilhelmsen , H. Filipsson Nyström , et al., “A Mechanistic Linkage Between Oral Lichen Planus and Autoimmune Thyroid Disease,” Oral Diseases 24, no. 6 (2018): 1001–1011. 10.1111/odi.12850 29500871

[10] A. Bobbio , P. Vescovi , L. Ampollini , and M. Rusca , “Oral Erosive Lichen Planus Regression After Thymoma Resection,” Annals of Thoracic Surgery 83, no. 3 (2007): 1197–1199. 10.1016/j.athoracsur.2006.08.024 17307497

[11] P. López‐Jornet , F. Parra‐Perez , and A. Pons‐Fuster , “Association of Autoimmune Diseases With Oral Lichen Planus: A Cross‐Sectional, Clinical Study,” Journal of the European Academy of Dermatology and Venereology 28, no. 7 (2014): 895–899. 10.1111/jdv.12202 23802853

[12] F. Lavaee and M. Majd , “Evaluation of the Association Between Oral Lichen Planus and Hypothyroidism: A Retrospective Comparative Study,” Journal of Dentistry (Shiraz, Iran) 17, no. 1 (2016): 38–42. PMC4771051 26966707

[13] M. Alikhani , P. Ghalaiani , E. Askariyan , Z. A. Khunsaraki , A. Tavangar , and A. Naderi , “Association Between the Clinical Severity of Oral Lichen Planus and Anti‐TPO Level in Thyroid Patients,” Brazilian Oral Research 31 (2017): e10. 10.1590/1807-3107BOR-2017.vol31.0010 28076499

[14] T. Zhang , F. Hou , D. Liu , et al., “Association of Hashimoto's Thyroiditis and Anti‐Thyroid Antibodies With Oral Lichen Planus: A Cross‐Sectional Study,” Frontiers in Immunology 13 (2022): 967988. 10.3389/fimmu.2022.967988 PMC9424685 36052085

[15] T. De Porras‐Carrique , P. Ramos‐García , M. Aguilar‐Diosdado , S. Warnakulasuriya , and M. Á. González‐Moles , “Autoimmune Disorders in Oral Lichen Planus: A Systematic Review and Meta‐Analysis,” Oral Diseases 29, no. 4 (2023): 1382–1394. 10.1111/odi.14127 35000260

[16] S. Piloni , F. Ferragina , I. Barca , E. Kallaverja , and M. G. Cristofaro , “The Correlation Between Oral Lichen Planus and Thyroid Pathologies: A Retrospective Study in a Sample of Italian Population,” European Journal of Dentistry 18 (2023): 510–516. 10.1055/s-0043-1772247 PMC11132789 37729935

[17] S. H. Lim , H. Kang , Y. W. Heo , W. S. Lee , and S. Lee , “Prevalence and Incidence of Comorbid Diseases and Mortality Risk Associated With Lichen Planopilaris: A Korean Nationwide Population‐Based Study,” Clinical and Experimental Dermatology 48, no. 11 (2023): 1230–1237. 10.1093/ced/llad235 37433080

[18] A. M. Radu , M. Carsote , C. Nistor , M. C. Dumitrascu , and F. Sandru , “Crossroads Between Skin and Endocrine Glands: The Interplay of Lichen Planus With Thyroid Anomalies,” Biomedicines 12, no. 1 (2023): 77. 10.3390/biomedicines12010077 PMC10813575 38255184

[19] J. B. Matthews , C. M. Scully , and A. J. C. Potts , “Oral Lichen Planus: An Immunoperoxidase Study Using Monoclonal Antibodies to Lymphocyte Subsets,” British Journal of Dermatology 111, no. 5 (1984): 587–595. 10.1111/j.1365-2133.1984.tb06629.x 6149762

[20] A. Kilpi , “Characterization of Mononuclear Cells of Inflammatory Infiltrates in Oral Tissues. A Histochemical and Immunohistochemical Study of Labial Salivary Glands in Sjögren's Syndrome and of Oral Lesions in Systemic Lupus Erythematosus and in Lichen Planus,” Proceedings of the Finnish Dental Society. Suomen Hammaslaakariseuran Toimituksia 84 Suppl 3, no. Suppl 3 (1988): 1–93. 3260669

[21] X. J. Zhou , P. B. Sugerman , N. W. Savage , L. J. Walsh , and G. J. Seymour , “Intra‐Epithelial CD8+ T Cells and Basement Membrane Disruption in Oral Lichen Planus,” Journal of Oral Pathology and Medicine 31, no. 1 (2002): 23–27. 10.1046/j.0904-2512.2001.10063.x 11896819

[22] J. Bogusławska , M. Godlewska , E. Gajda , and A. Piekiełko‐Witkowska , “Cellular and Molecular Basis of Thyroid Autoimmunity,” European Thyroid Journal 11, no. 1 (2022): e210024. 10.1530/ETJ-21-0024 PMC9142813 34981746

[23] P. G. Arduino , D. Karimi , F. Tirone , et al., “Evidence of Earlier Thyroid Dysfunction in Newly Diagnosed Oral Lichen Planus Patients: A Hint for Endocrinologists,” Endocrine Connections 6, no. 8 (2017): 726–730. 10.1530/EC-17-0262 PMC5670272 29101247

[24] D. Li , J. Li , C. Li , Q. Chen , and H. Hua , “The Association of Thyroid Disease and Oral Lichen Planus: A Literature Review and Meta‐Analysis,” Frontiers in Endocrinology 8 (2017): 310. 10.3389/fendo.2017.00310 PMC5684121 29170653

[25] T. Zhou , D. Li , Q. Chen , H. Hua , and C. Li , “Correlation Between Oral Lichen Planus and Thyroid Disease in China: A Case‐Control Study,” Frontiers in Endocrinology 9 (2018): 330. 10.3389/fendo.2018.00330 PMC6015892 29967591

[26] P. A. Amato‐Cuartas , A. E. Tabares‐Quintero , L. F. Vélez‐Jaramillo , et al., “Coexistence of Thyroid Disease and Oral Lichen Planus in a Colombian Population,” Acta Odontologica Latinoamericana 32, no. 2 (2019): 71–74. 31664296

[27] N. Brankov , R. Z. Conic , N. Atanaskova‐Mesinkovska , M. Piliang , and W. F. Bergfeld , “Comorbid Conditions in Lichen Planopilaris: A Retrospective Data Analysis of 334 Patients,” International Journal of Women's Dermatology 4, no. 3 (2018): 180–184. 10.1016/j.ijwd.2018.04.001 PMC6116820 30175224

[28] K. Phan , O. Charlton , and S. Smith , “Lichen Planopilaris and Thyroid Disease: Systematic Review and Meta‐Analysis,” Australasian Journal of Dermatology 60 (2019): 74.

[29] A. Mohiti , M. Sadrabad , R. Ghorbani , R. Fallah , N. Tabavar , and A. Pedram , “Concurrent Incident of Oral Lichen Planus and Hyperthyroidsm and Hypothyroidism,” Koomesh 21 (2019): 41–45.

[30] M. Vehviläinen , A. Salem , M. Y. Asghar , T. Salo , and M. Siponen , “No Detection of TSH or TSHR in Oral Lichen Planus Lesions in Patients With or Without Hypothyroidism,” Acta Odontologica Scandinavica 78, no. 5 (2020): 337–344. 10.1080/00016357.2020.1720798 32031461

[31] P. Toossi , A. Sebti , and F. Sarvghadi , “Thyroid Diseases and Lichen Planopilaris: A Case Control Study,” Iranian Journal of Dermatology 18 (2015): 104–107.

[32] H. Babahosseini , S. Tavakolpour , H. Mahmoudi , et al., “Lichen Planopilaris: Retrospective Study on the Characteristics and Treatment of 291 Patients,” Journal of Dermatological Treatment 30 (2019): 598–604. 10.1080/09546634.2018.1542480 30411987

